# Computational Fluid Dynamics Simulations of Mitral Paravalvular Leaks in Human Heart

**DOI:** 10.3390/ma14237354

**Published:** 2021-11-30

**Authors:** Krzysztof Wojtas, Michał Kozłowski, Wojciech Orciuch, Łukasz Makowski

**Affiliations:** 1 Faculty of Chemical and Process Engineering, Warsaw University of Technology, Warynskiego 1, 00-645 Warsaw, Poland; wojciech.orciuch@pw.edu.pl (W.O.); lukasz.makowski.ichip@pw.edu.pl (Ł.M.); 2 Department of Cardiology and Structural Heart Diseases, Medical University of Silesia, Ziolowa 45/47, 40-635 Katowice, Poland; mkozlowski303@gmail.com

**Keywords:** paravalvular leak, hemolysis, hemodynamics, computational fluid dynamics

## Abstract

In recent years, computational fluid dynamics (CFD) has been extensively used in biomedical research on heart diseases due to its non-invasiveness and relative ease of use in predicting flow patterns inside the cardiovascular system. In this study, a modeling approach involving CFD simulations was employed to study hemodynamics inside the left ventricle (LV) of a human heart affected by a mitral paravalvular leak (PVL). A simplified LV geometry with four PVL variants that varied in shape and size was studied. Predicted blood flow parameters, mainly velocity and shear stress distributions, were used as indicators of how presence of PVLs correlates with risk and severity of hemolysis. The calculations performed in the study showed a high risk of hemolysis in all analyzed cases, with the maximum shear stress values considerably exceeding the safe level of 300 Pa. Results of our study indicated that there was no simple relationship between PVL geometry and the risk of hemolysis. Two factors that potentially played a role in hemolysis severity, namely erythrocyte exposure time and the volume of fluid in which shear stress exceeded a critical value, were not directly proportional to any of the characteristic geometrical parameters (shape, diameters, circumference, area, volume) of the PVL channel. Potential limitations of the proposed simplified approach of flow analysis are discussed, and possible modifications to increase the accuracy and plausibility of the results are presented.

## 1. Introduction

Paravalvular leaks (PVLs) following surgical valve replacement are relatively common, affecting approximately 22% of patients with prosthetic mitral valves at the time of discharge after surgery [1]. In some cases, PVLs can cause hemolysis, which may develop into a serious problem that requires repeated blood transfusions and warrants interventional treatment. Nowadays, the percutaneous closure of PVLs is the procedure of choice whenever feasible. Currently, there is no dedicated device approved by the U.S. Food and Drug Administration for this procedure [2,3], although ongoing clinical trials intend to fill this gap (e.g., see ClinicalTrials.gov identifier NCT04489823). As a consequence, only off-label devices, such as umbrella devices [4] or vascular occluding devices [5], provide a therapeutic option. According to current data, clinically significant hemolysis is the main indication for PVL closure in 10–20% of cases [6]. Successful intervention is associated with lower cardiac mortality and decreased severity of hemolysis [7].

Unfortunately, percutaneous PVL closure is not always successful. Persistent hemolytic anemia after an attempted PVL closure is a predictor of poor survival [2]. For unclear reasons, hemolysis worsens in 19.7% of patients after transcatheter closure of PVLs [8]. Such an adverse outcome can occur despite satisfactory results observed in periprocedural echocardiographic examinations. To the best of our knowledge, no research has been conducted to determine the mechanisms of hemolysis following PVL closure.

The pathogenesis of hemolysis in patients with PVLs seems to be multifactorial, and the current data indicates that the high shear stress forces that affect the erythrocytes passing through PVL channels may be crucial. Since the nature of blood flow is complex, and PVL channel geometries vary significantly, it has not been possible to create comprehensive models of the flow for the in vitro assessment of shear stress distribution.

Nowadays, specialized computer software can be used to perform simulations. Computational fluid dynamics (CFD) is a numerical modeling method that involves solving equations pertaining to the differential balance of mass, momentum, and energy under the assumption of medium continuity for a small section of fluid known as a computational cell. Application of this method allows to create flow patterns and to precisely assess shear stress distribution in fluids. CFD has been used extensively in medicine for analyzing wall shear stresses in coronary arteries and for pre-procedural planning of carotid endarterectomies [9,10,11,12,13].

Attempts have been made to utilize CFD to create simulations of blood flow through PVL channels. A group of researchers distinguished several patterns of flow through PVLs and correlated these patterns with elevated biochemical markers of hemolysis [14]. However, this study had certain limitations: the researchers analyzed only eight PVLs, assumed that the pressure gradient between the cardiac chambers connected by the PVL was constant, did not consider the geometry of the PVL channel, and did not examine whether the type of prosthesis implanted would influence the severity of hemolysis. In addition, they were not able to assess how the percutaneous closure of PVLs using various types of devices would change the flow patterns, since such procedures were not performed at that time. These limitations could not be overcome in the past due to lack of sophisticated computer equipment and three-dimensional imaging with an adequate spatial resolution. Nowadays, data obtained from echocardiography and computed tomography scanners can be used to create exact models of both the PVL channels and surrounding heart structures [15,16], and these models can be used for the assessment of flow without any geometrical assumptions or simplifications.

In this study, we created a simplified model of the left ventricle (LV) based on a computed tomographic examination of a patient with a mitral PVL. Literature provides numerous examples that discuss the use of dynamic deformation of the LV in the blood flow simulations both in healthy and infarcted LVs, as well as in subjects with native, bioprosthetic, healthy, and regurgitant mitral valves, or with left ventricular assist devices (see, e.g., [17,18,19,20,21]). However, preparation of the heart geometry and calculations themselves are much more complex in the abovementioned examples than in the case of a simplified, universal LV geometry. Thus, in our opinion, a simplified approach where only the PVL shape needs to be reproduced exactly, has potential to gain a widespread application in PVL diagnostics and treatment. In addition, the main aim of this work was to evaluate whether such a simplified approach would make it possible to predict flow characteristics that would serve as a risk indicator of hemolysis, and to determine the influence of relative differences between PVL geometries on this risk. It should also be mentioned that such an approach (simplified geometry) is commonly found in the literature and allows to obtain credible results during CFD modeling of physiological systems and conditions [22].

Calculations were performed for a situation in which the mitral valve was completely closed during chamber contraction. As a result, only backward flow occurred through the PVL from the left ventricle to the left atrium. The purpose of performed calculations was to determine shear stress values affecting erythrocytes passing through a mitral PVL and ascertain whether such stress could result in hemolysis. In order to properly validate simulations, it would be required to create a PVL model based on real-life imaging data from a patient with a mitral PVL, and at the same time analyze the presence and severity of hemolysis in the same subject. However, we believe that the influence of such complex factors should be excluded in the initial phase of research. Therefore, to validate the results, literature data regarding critical shear stress values should be utilized (considering discrepancies in available values amongst published articles). The critical value of the tangential stress ranges from 150 Pa (the value obtained using a rotary rheometer for an exposure time of about 1 min) to 400 Pa, 600 Pa, or even 800 Pa (values obtained in a turbulent stream from a nozzle for an exposure time of 1 ms [23,24,25,26,27]. Although several models for hemolysis index are available in the literature, their validity range remains mostly unclear and no universally applicable formulation has been given so far [28]. A value of 150 Pa is recommended and used as an indicator of hemolysis in at least several studies [29,30,31,32]. In this work, however, 300 Pa was taken as the critical shear stress value [27], due to the expected shorter exposure times and the potentially higher shear resistance of the patient’s erythrocytes. Four PVL geometries at different leakage rates were studied (28 cases in total).

## 2. Numerical Method

In this study, intraventricular blood flow was simulated using the commercial CFD software ANSYS Fluent 2021R1. Blood was assumed to be a Newtonian fluid with viscosity μ = 3.45 × 10^−3^ Pa s and density ρ = 1060 kg m^−3^. Its motion was governed by the three-dimensional Navier–Stokes equations for a viscous incompressible flow:

(1) $$\frac{\partial u_{i}}{\partial t}+\frac{\partial \left(u_{i}u_{j}\right)}{\partial x_{j}}=-\frac{1}{\rho }\frac{\partial p}{\partial x_{i}}+\nu \frac{\partial }{\partial x_{j}}\left(\frac{\partial u_{i}}{\partial x_{j}}\right)$$

(2) $$\frac{\partial u_{i}}{\partial x_{i}}=0$$

To solve the Navier–Stokes equations, a *k-ω* shear stress transport (SST) turbulence model was used, since the *k-ω* turbulence model is proved to yield the most accurate results in cases of physiological systems [33]. The model is based on the Boussinesq hypothesis, which assumes that the Reynolds stress tensor, $\tau_{ij}$, can be related to the mean velocity gradients by a turbulent (eddy) viscosity $\mu_{t}$:

(3) $$\tau_{ij}=-\rho \bar{u_{i}u_{j}}=\mu_{t}\left(\frac{\partial U_{i}}{\partial x_{j}}+\frac{\partial U_{j}}{\partial x_{i}}\right)-\frac{2}{3}\left(\rho k+\mu_{t}\frac{\partial U_{i}}{\partial x_{i}}\right)\delta_{ij}$$

where ρ is the fluid density, $U_{i}$ is the velocity vector component, and k is the turbulent kinetic energy. The turbulent viscosity is proportional to k and is computed from:

(4) $$\mu_{t}=\rho \min\left(\frac{k}{\omega },\frac{ak}{S\tanh\left(\phi^{2}\right)}\right)$$

(5) $$\phi =\max\left(\frac{2\sqrt{k}}{0.09\omega y},\frac{500\nu }{y^{2}\omega }\right)$$

where ω is the turbulence frequency, a is the model constant, S is the strain rate magnitude, ν is the kinematic viscosity, and y is the wall distance.

Shear stress magnitude within the fluid (in the interior of the system) can be calculated based on the available turbulence properties using the following expression [34]:

(6) $$\tau =\mu_{eff}\sqrt{2S_{ij}S_{ij}}=\left(\mu +\mu_{t}\right)\sqrt{2S_{ij}S_{ij}}$$

where $\mu_{eff}$ is the effective viscosity and $S_{ij}$ is the strain rate tensor.

In the simulations, the numerical mesh used for the LV was created in ANSYS Fluent Meshing 2021R1 and consisted of approximately 500,000 poly-hexcore cells with an average size of 1 mm. The boundary layer on each wall consisted of 10 elements and was generated using the smooth-transition method with an expansion ratio of 1.15. This boundary layer ensured that the $y^{+}$~1 condition could be satisfied for the highest tested blood flow rate. In the zone surrounding the channel between the LV and left atrium (LA), i.e., in the region with the highest energy dissipation rate and strain rate values, the mesh was of higher density with cell sizes of 0.5 mm or smaller. Mesh independence was checked using both the average turbulent energy dissipation rate in the system and the average wall shear stress at the walls. Further mesh refinement did not produce any differences in the results, i.e., both these quantities were constant (approximately 1–2% difference between meshes).

The coupled solver in Fluent was used for the pressure-velocity coupling and second-order discretization schemes were used for all variables to minimize numerical diffusion effects. Computations were regarded to have satisfactorily converged when the total normalized residuals were smaller than 10^−6^, and a constant value of the average wall shear stress was achieved on each wall.

## 3. Simulation Setup

**Figure 1:**
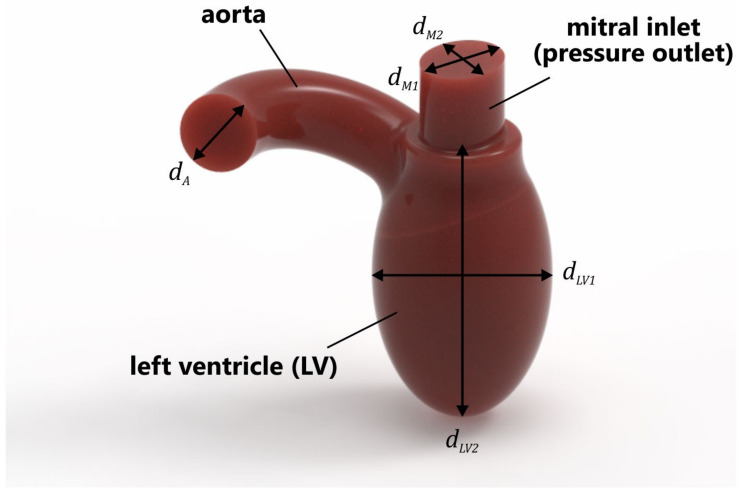
Simplified geometry of LV.

In this study, a simplified model of the LV was used (Figure 1). The volume of the ventricle was 87 mL, which was assumed to be the average of the diastolic peak volume (118 mL) and end-systolic volume (56 mL) [20]. The geometrical parameters of the LV were as follows: $d_{M1}$ = 24 mm, $d_{M2}$ = 20 mm, $d_{A}$ = 20 mm, $d_{LV1}$ = 45 mm, and $d_{LV2}$ = 76.5 mm. In the simulations, only the systolic phase was considered, i.e., the mitral valve was fully closed, and flow occurred only through the PVL. This caused blood to flow backward into the left atrium; in our case, into the mitral inlet (pressure outlet), which also imitated the LA in this study.

**Figure 2:**
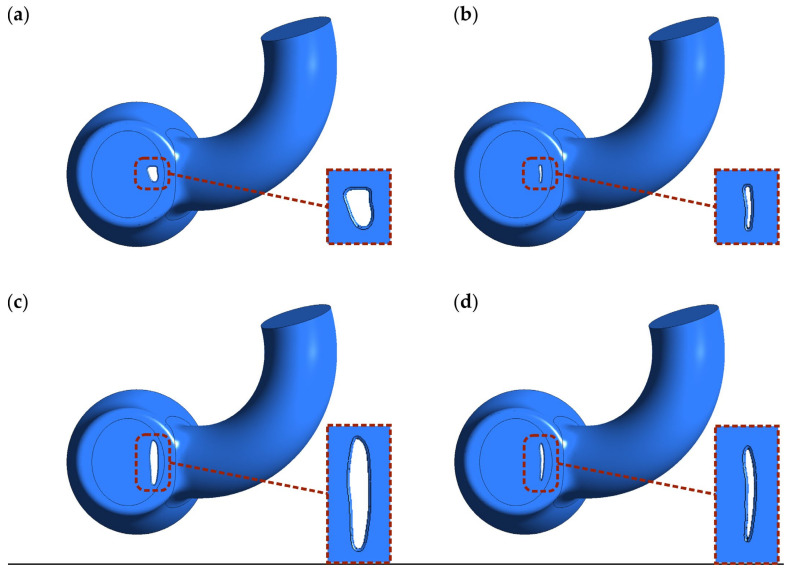
Shape and location of PVL channels around the mitral valve. Channel height: 3 mm. CSA: (**a**) 7.9 mm^2^; (**b**) 1.5 mm^2^; (**c**) 18.5 mm^2^; (**d**) 5.0 mm^2^.

Four different PVL geometries were studied in this work, as shown in Figure 2. The main characteristics differentiating the geometries were the cross-sectional area (CSA) and shape. The studied geometries were generated based on the imaging data [8,35,36,37], and could be considered as representative of clinical cases of real-life PVLs. The wide channels (Figure 2a,c) were created to reproduce untreated PVLs, while the narrower channels (Figure 2b,d) were meant to represent PVLs that were not fully closed after the percutaneous closure. Hereafter, the different cases are referred to as PVL I, PVL II, PVL III, and PVL IV with CSAs of 7.9, 1.5, 18.5, and 5.0 mm^2^, respectively. The height of each channel was 3 mm. PVL walls were perpendicular to the upper wall of the LV, and their connection was rounded; the radius was equal to 0.3 mm. Vertical cross-sections of the analyzed geometries were presented in Figures 7 and 10, while the overall shape was more clearly visible in Figure 8.

**Figure 3:**
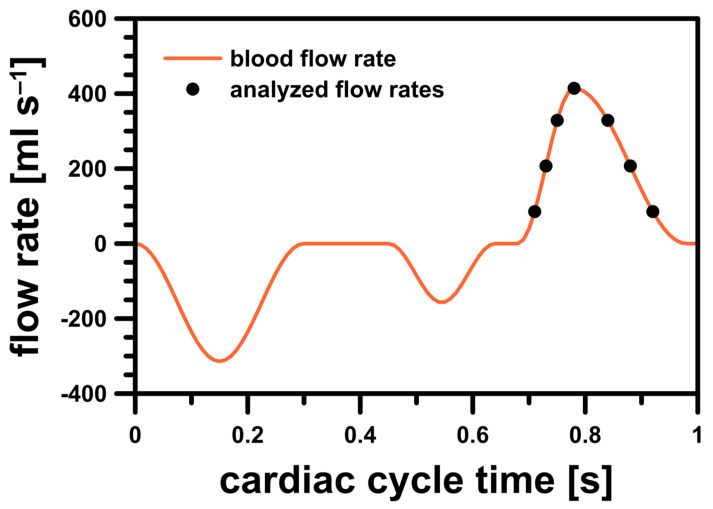
Blood flow rate profile for LV model.

The simulations were conducted in an unsteady-state and aimed to investigate whether the risk of hemolysis could be assessed by checking the maximum wall shear stress and strain rate values in the PVL region. A time-step value equal to 1 ms was used for each studied case. The blood volume flow rate profile during the cardiac cycle was based on a model found in the literature [20] (Figure 3). In this profile, a heart rate of 60 beats per minute was assumed, i.e., one full cardiac cycle took exactly 1 s. Systole phase lasted exactly 0.3 s and began at 0.68 s of the cardiac cycle.

To properly analyze blood flow differences in the simulations during systole, seven representative discrete points on the blood flow rate profile were selected. In order to check in which stage of the systole the risk of hemolysis was higher (if at all), the selected points were analyzed in two cases: during the acceleration and deceleration of left ventricular contraction. The studied flow rates were illustrated in Figure 3 and their exact values were listed in Table 1.

**Figure 4:**
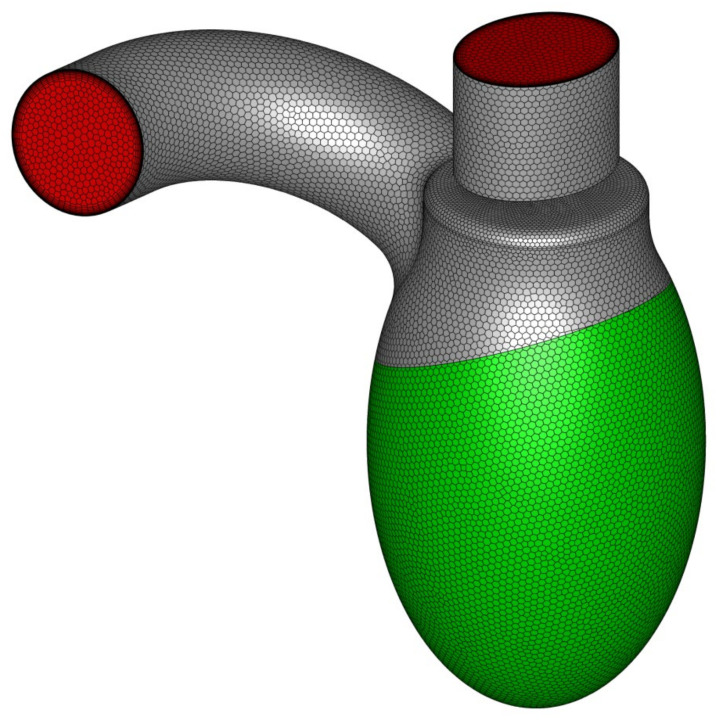
LV geometry with marked boundary condition types: green represents mass flow inlet, whereas red represents pressure outlet.

To represent LV motion, the most obvious solution was to use a dynamic/deforming mesh. Unfortunately, the preparation phase was a complex and time-consuming process, not to mention the increased computation time that made such a solution impractical in day-to-day medical diagnostics. For this reason, a different approach was used—instead of describing the exact wall movement, flow characteristics (induced mass flux) over time were described using a mathematical expression [20]. Equivalent boundary conditions were applied in the computations: a mass flow inlet condition for the LV side wall and a pressure outlet condition for both the aortic and mitral outlets (Figure 4). The blood flow rate profile varied over time and was defined as a user defined function. The mass flux was delivered through the side wall of the left ventricle (green surface in Figure 4). The stream was distributed evenly on the whole surface and the inlet condition was set as “normal to boundary” (at each facet).

The left atrial pressure during systole under normal conditions was approximately 6 mmHg relative to the atmosphere [38]. In the pathological setting of a PVL, a higher value could be expected. In this study, based on the data available in the literature for patients following LA catheterization, the mean LA pressure was assumed to be 23 mmHg (3066.4 Pa) [39]. Mean systolic arterial blood pressure for the same patient group was equal to 102 mm Hg (13,598.8 Pa) [39], and this value was used in the simulation for both aortic outlet (outlet) and LV inlet (mass flow inlet). Therefore, the pressure difference between the LA and LV in the simulations was 79 mmHg (10,532.4 Pa).

## 4. Results and Discussion

**Figure 5:**
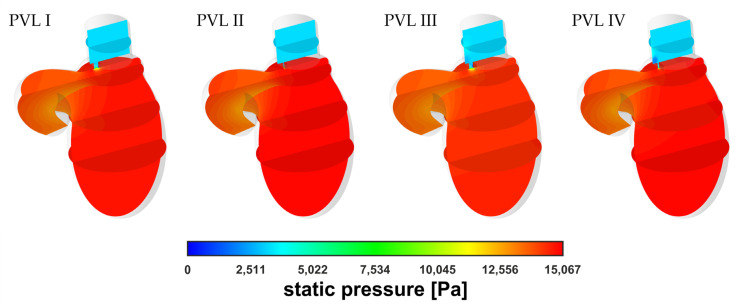
Contours of static pressure at $\dot{m}$ = 0.439 kg s^−1^.

The simulation results showed no significant differences in terms of pressure distribution among all geometrical variants at equal blood mass flow rates, $\dot{m}$. A comparison of the static pressure distribution for different PVL geometries at $\dot{m}$ = 0.439 kg s^−1^ was shown in Figure 5. The pressure buildup visible in the LV was related to the increasing flow resistance (pressure drop) in the aorta at higher flow rates. However, in the most important region, with respect to PVLs, i.e., the channel, the results were comparable regardless of PVL geometry. The lack of significant pressure changes in the LA among different phases of systole was related to the constant pressure applied at both the mitral and aortic outlet boundaries for all flow rates.

**Figure 6:**
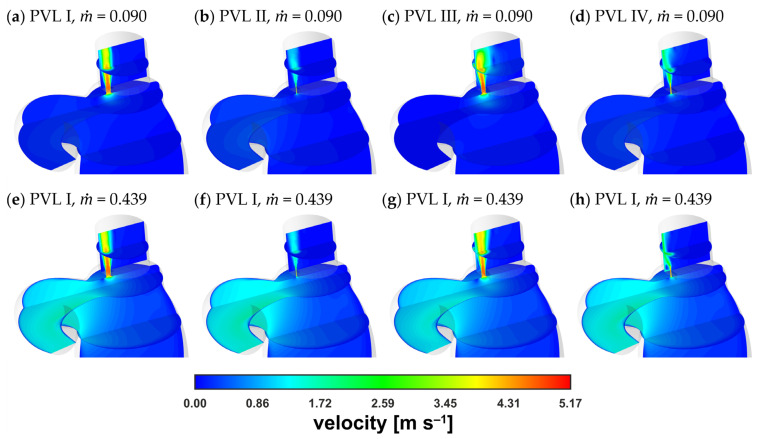
Contours of velocity magnitude: (**a**–**d**) $\dot{m}$ = 0.090 kg s^−1^; (**e**–**h**) $\dot{m}$ = 0.439 kg s^−1^.

Figure 6 showed comparison of the velocity distribution in the LV and a similar tendency regarding pressure distribution could be observed. The velocity distribution in the aorta was comparable at equal flow rates, irrespective of PVL geometry. The largest differences in the distribution were observed in the channel and above in the LA. The main differentiating factor here was the shape (cone) of the velocity profile, whereas the maximum blood velocity was similar regardless of the variant (geometry and flow rate).

**Figure 7:**
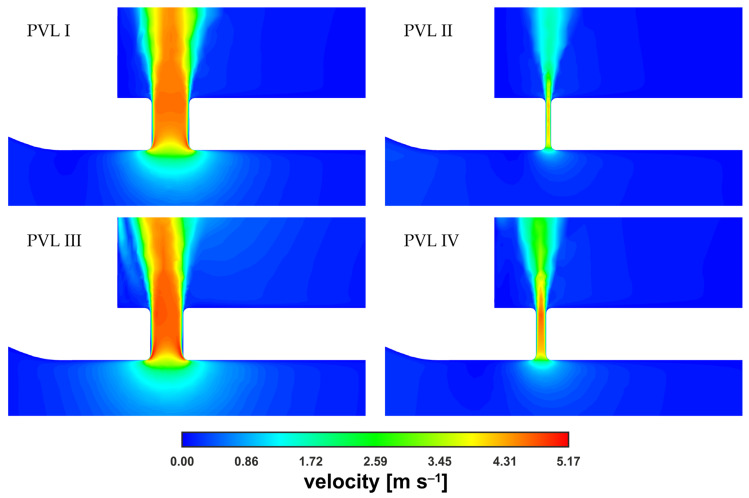
Contours of velocity magnitude inside PVLs at $\dot{m}$ = 0.090 kg s^−1^.

A magnified view of the PVL region at $\dot{m}$ = 0.090 kg s^−1^ was obtained (Figure 7) to better illustrate the similarity between velocity magnitude profiles. The results were presented for the same vertical plane as that considered in Figure 6 (the plane cuts PVLs in half). The cone shape depended mostly on the PVL width and that dependence on the PVL width was also true for the length of the high-velocity zone.

The velocity magnitude distribution along the PVL axis showed a rapid increase just past the channel entrance. The evolution of the velocity profile was visible (with the transition to a fully developed profile), reaching maximum values of approximately 5 m s^−1^. The profile was rather typical for a duct flow, and observed irregularities in the velocity distribution, i.e., a noticeable asymmetry, could be attributed to the transient nature of the flow. To better show the small differences in maximum velocities, the characteristic values in the channel were listed in Table 2. In the table, $u_{CP}$ was the velocity at the exact center point (both horizontally and vertically) of the PVL section seen in Figure 7, and $u_{AVG}$ was the mass-weighted average velocity over the entire PVL volume (PVL volumes were listed in Table A1 in the Appendix A).

As shown in Table 2, as well as in Figure 6 and Figure 7, for all the studied PVL geometries, the maximum velocity magnitude values were similar, which could be attributed to the constant pressure applied at the boundaries. It can be argued that during the cardiac cycle, the blood pressure changed in both the LV and LA together with the blood flow rate, making it inappropriate to define such boundary conditions. Consequently, the difference between the blood pressures at the aortic and mitral outlets should be smaller for cases with lower flow rates. However, information on left atrial pressure fluctuations during a cardiac cycle (in the presence of a PVL) was not available in the literature. Therefore, the application of a simple mathematical expression at the boundaries was not feasible. Moreover, an advanced analysis was not within the scope of this study since the study aimed to assess the usefulness of a simplified approach to model the PVL condition.

Since the velocity and pressure distributions failed to provide conclusive insights into the risk of hemolysis, the shear stress magnitude, τ, and wall shear stress, $\tau_{w}$, could be considered as better risk indicators. The wall shear stress, which was calculated at the walls, was a variable available in Fluent and could be accessed freely. The shear stress magnitude in the interior of the system was obtained using Equation (6).

**Figure 8:**
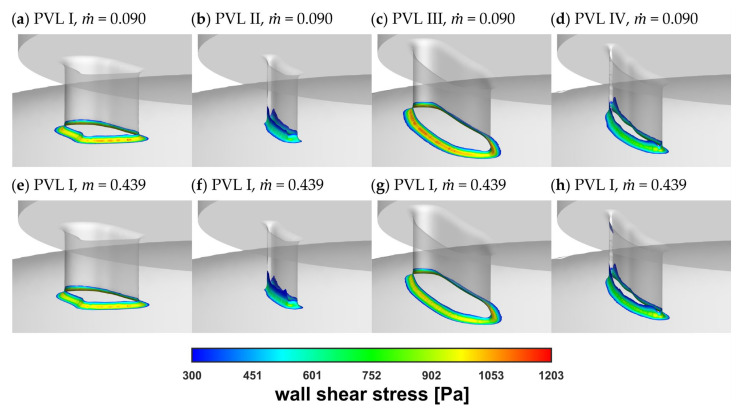
Wall shear stress at channel walls: (**a**–**d**) $\dot{m}$ = 0.090 kg s^−1^; (**e**–**h**) $\dot{m}$ = 0.439 kg s^−1^.

Figure 8 showed the wall shear stress contours at the channel walls. In the figure, the colored areas corresponded to stress values larger than 300 Pa (safe level), above which blood hemolysis could occur [27].

**Figure 9:**
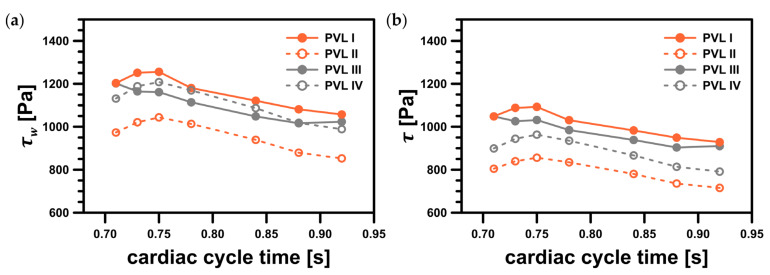
Maximum shear stress values in the considered system: (**a**) maximum wall shear stress, $\tau_{w}$; (**b**) maximum shear stress magnitude, τ. Both variables represent values in a specific point; facet maximum for wall shear stress and cell maximum for shear stress magnitude.

The safe level was exceeded only in a small area around the channel entrance for all studied PVL geometries, making the shape of the channel entrance another key factor affecting the probability of hemolysis. The maximum wall shear stress values, presented in Figure 9a, tended to increase in the acceleration phase of LV contraction and to decrease in the deceleration phase. Moreover, the maximum wall shear stress was higher at the beginning of the systole. This was related to the more rapid change in the blood flow rate in the first 0.1 s of the systole—in the profile used, it took 0.1 s to reach maximum flow rate (0.439 kg s^−1^), and then 0.2 s until the flow stopped. The shear stress magnitude inside the channel (Figure 9b) showed similar tendencies, with slightly smaller values than those at the channel walls. However, this observation was expected, since shear stress decreased away from the walls. Higher shear stress values obtained for PVL I and PVL III could be observed, and this may indicate that wider channels were more prone to hemolysis. The exact values presented in Figure 9 were listed in Appendix A in Table A1.

**Figure 10:**
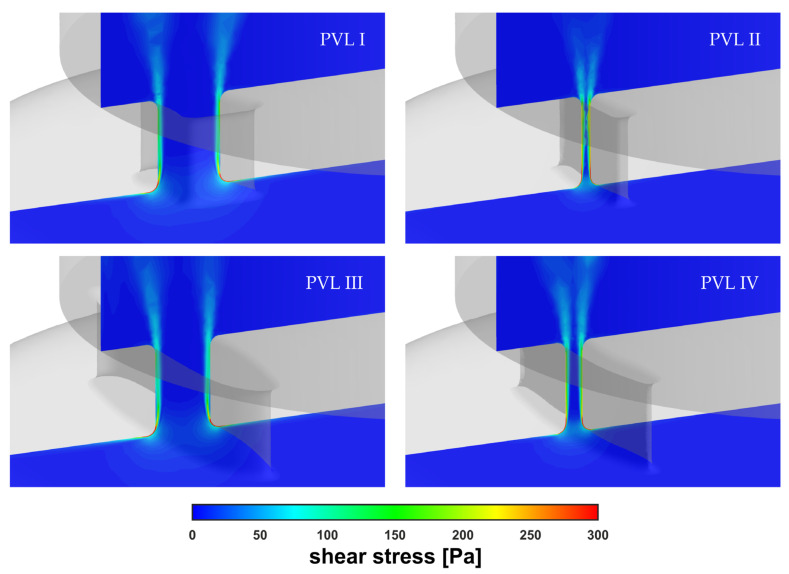
Contours of shear stress inside PVLs at $\dot{m}$ = 0.439 kg s^−1^.

Figure 10 showed the shear stress profiles inside the channels at $\dot{m}$ = 0.439 kg s^−1^. The red-colored regions indicated the areas where the shear stress exceeded the value of 300 Pa. The highest shear stress values were found at the channel entrance, which was the same region shown in Figure 8. However, the most important observation was the existence of a non-negligible, very small volume, defined here as $V_{300}$, wherein the safe shear stress level was exceeded. The locations of the areas with the highest stress values coincide with the literature data. In [40], the authors performed numerical simulations of blood flow in hemodialysis cannulas. They clearly observed that the highest stresses (shear rates of >50,000 s^−1^ in their case) occurred in a small region near the rapid velocity change, caused by the geometry modification, and inside the channel lumen as the internal vortex developed. Authors indicated that the velocities and shear rates at these locations showed that the geometry of channels had influence on the levels of hemolysis.

**Figure 11:**
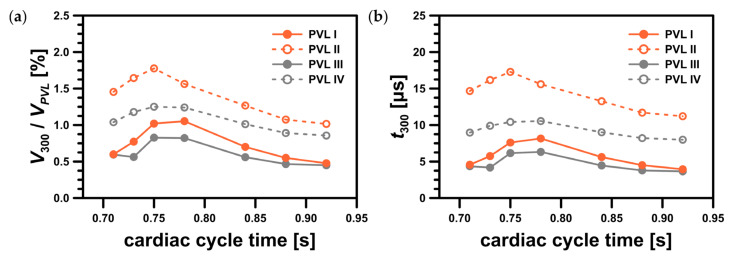
Characteristics of region with shear stress value greater than 300 Pa: (**a**) ratio of the region volume, $V_{300}$, to the PVL volume, $V_{PVL}$; (**b**) residence time in the region, $t_{300}$.

For PVL II and PVL IV, the high shear volume propagated along the channel walls making it a larger fraction of the total channel volume, $V_{PVL}$, than for PVLs I and III (Figure 11a). In this figure, the ratio of $V_{300}$ to $V_{PVL}$ expressed in what proportion of the PVL volume the critical shear stress value was exceeded. This showed that the risk of hemolysis was high for all studied PVL variants, and the data presented in Figure 9 indicated that the wider the PVL, the more severe the hemolysis. This was not necessarily true, due to reasons that have not yet been fully elucidated, hemolysis worsens in 19.7% of patients after transcatheter closure of PVLs [8]. This strongly suggested that there was another possible factor responsible for the worsening of hemolysis.

Although the critical value of the shear stress related to hemolysis was defined, it was unlikely that it was a sufficient criterion to assess the severity of hemolysis in relation to the PVL condition. The volume of the regions where the critical shear stress value was exceeded and the duration of the exposure of red blood cells to the critical shear stress should also be acknowledged. Let’s consider here the residence time in a region where the shear stress exceeds 300 Pa, $t_{300}$. To estimate the residence time, we can use the volume of this region, $V_{300}$, and the mass flow rate through the PVL, $\dot{m_{PVL}}$, both of which can be exported from CFD software:

(7) $$t_{300}=V_{300}\frac{\rho }{\dot{m_{PVL}}}$$

The results of such a simple analysis based on the average flow rates through the PVLs were presented in Figure 11b (for exact values see Table A1 in the Appendix A).

Since significant hemolysis was not observed clinically in all mitral PVLs, the volume ($V_{300}$) and residence time ($t_{300}$) in the region where the critical shear stress value was exceeded were potential additional factors that might play a role in hemolysis severity. Dependence of hemolysis probability on these two parameters had never been tested in a clinical scenario, but we hypothesized that the larger the volume, $V_{300}$, and the longer the residence time, $t_{300}$, the more significant degree of hemolysis should be expected. Based on Figure 11, it seemed there was no simple relationship between the “critical” volume and the CSA, or total volume of a PVL. For example, the largest $V_{300}$ value was observed for PVL III (both largest CSA and total volume). On the other hand, in the case of PVLs II and IV, which had relatively small CSAs, smaller $V_{300}$ volumes were observed, but the time of erythrocytes exposure, $t_{300}$, was longer. Moreover, even though the maximum shear stress values were higher for PVLs I and III (wide PVLs), the high shear region relative volume was larger for PVLs II and IV (narrow PVLs). Since the analyzed PVL geometries differ significantly in terms of key parameters (circumference, CSA, total volume, wall area), it was unlikely that the residence time, $t_{300}$, was related to these parameters in a simple way. In clinical practice, various PVL shapes were encountered: oval, round, slit-like, or crescent. A possible way to mathematically describe PVL shapes was to use both the maximal and minimal dimensions of a PVL. Performing such measurements, however, was not standardized and the results were not widely reported. Differences in shapes might explain why for a narrow PVL (PVL IV) the volume, wherein the critical shear stress value was exceeded, was similar to a wide PVL (PVL I) despite PVL I having both larger CSA and volume. Since shape appeared to be an independent variable that affected the probability of hemolysis, and simultaneously was difficult to define mathematically, further research was necessary to determine these relationships.

Since the critical value of shear stresses depended on the exposure time (the longer the exposure time, the lower the critical value) [25], it seemed that more blood cells were destroyed in relatively narrow channels. This agreed well with the literature data, which indicated that small residual PVL leaks, that may still be present after transcatheter closure of PVLs, can be responsible for the worsening of hemolysis [8]. To the best of our knowledge, this work was the first example where CFD was applied for blood flow simulation through mitral PVLs to provide detailed data of critical process parameters affecting the risk of hemolysis—maximum shear stress values at the walls and in fluid, volume of the region where critical shear stress value was exceeded and exposure time of erythrocytes to this critical shear stress. Although this simple study provided valuable insights into the factors responsible for hemolysis, to quantitatively assess the risk and magnitude of hemolysis, it is necessary to conduct more complex analyses, e.g., analysis that considers population balance to describe the probability of erythrocyte death. This is a good indication of the direction that future work should follow.

**Figure 12:**
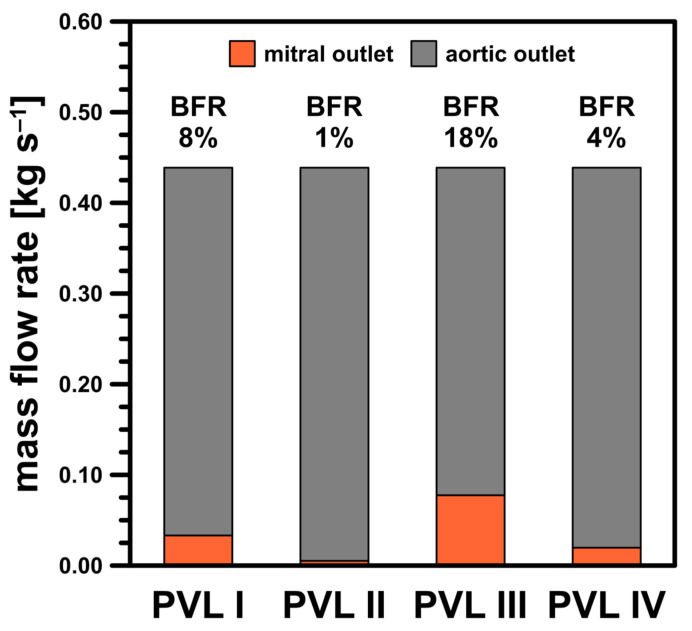
Mass balance for studied PVL geometries at $\dot{m}$ = 0.439 kg s^−1^.

Finally, the mass balance in the LV should be addressed. Figure 12 presented the mass balance at $\dot{m}$ = 0.439 kg s^−1^ as the combined mass flow rates through both the aortic and mitral pressure outlets. Here, an auxiliary parameter, the backflow ratio (BFR), was defined as the ratio of the flow at the mitral outlet to that at the inlet stream. As the CSA increased, the BFR values increased, as expected, due to higher flow resistance in the channel at high flow rates (Figure 5). This was another risk factor regarding severity of PVL condition, i.e., the higher the BFR, the higher the severity. However, since the present results were obtained using average pressure values at the outlets, the obtained values should be considered as illustrative only. This ultimately leads to the conclusion that a complete description of a PVL-affected cardiac cycle with more accurate BFRs requires further, more comprehensive analysis, as previously mentioned in this section.

## 5. Summary

Currently, cardiologists and cardiac surgeons are uncertain about the mechanism of hemolysis in patients with PVLs. To clarify this mechanism, a simplified modeling approach, involving transient CFD simulations, was employed in this study to investigate the risk of hemolysis accompanying a PVL condition. For this purpose, four PVL geometries with different CSAs (1.5–18.5 mm^2^) were studied at seven discrete blood flow rates (85.3–414 mL s^−1^). In each case, numerical modeling indicated the areas where the shear stress at the channel walls significantly exceeded 300 Pa (up to 1200 Pa), thus having a disruptive effect on blood cells. Since hemolysis is not present clinically in all patients with PVLs, it can be assumed that a certain volume of blood must be exposed to shear stress greater than 300 Pa to cause significant hemolysis.

In this study, CFD allowed us to predict the levels of shear stress inside a heart affected by a PVL, and thus provided information about hemolysis probability. Moreover, the modeling approach allowed to distinguish different PVL geometries in terms of parameters affecting the risk and severity of hemolysis. However, it is important to remember that several simplifications were adopted in this study: a simplified LV geometry, PVL channels were straight and without any bends, the treatment of blood as a Newtonian fluid, and the assumption of constant pressure values at the boundaries. Avoiding these assumptions was not entirely possible since the insufficient (or even the complete lack of) literature data. For this reason, in our future works we plan to expand the scope of this study to address these issues. This will allow us to meet the long-term goal of this research; to develop a “risk map” for hemolysis in order to determine the relationship between distinctive PVL dimensions (and associated blood flow) and the likelihood of hemolysis. This tool is intended to assist clinical practitioners in day-to-day diagnostics of PVLs and as an additional indicator of eligibility for percutaneous closure. Nonetheless, the usefulness of this relatively simple and easy-to-implement modeling approach can be assessed positively, considering that it provides valuable insights into the PVL condition.

## Figures and Tables

**Table 1:** Analyzed blood volume and mass flow rates.

Cardiac Cycle Time, t, s	Blood Volume Flow Rate, mL s^−1^	Mass Flow Rate, $\dot{m}$, kg s^−1^
0.71	85.3	0.090
0.73	207.0	0.219
0.75	328.7	0.348
0.78	414.0	0.439
0.84	328.7	0.348
0.88	207.0	0.219
0.92	85.3	0.090

**Table 2:** Characteristic velocity magnitude values inside PVLs.

Geometry	Mass Flow Rate, $\dot{m}$, kg s^−1^	Velocity Magnitude at PVL Center Point, $u_{CP}$, m s^−1^	Mass-Weighted Average Velocity Inside PVL, $u_{AVG}$, m s^−1^
PVL I	0.090	4.622	3.999
	0.439	4.648	3.951
PVL II	0.090	3.915	2.984
	0.439	3.973	3.018
PVL III	0.090	4.770	4.155
	0.439	4.697	3.977
PVL IV	0.090	4.524	3.484
	0.439	4.600	3.540

## Data Availability

The data presented in this study are contained within the article.

## Appendix A

**Table A1:** Data used to prepare figures.

Geometry	Cardiac Cycle Time t, s	Mass Flow Rate $\dot{m}$, kg s^−1^	Maximum Wall Shear Stress $\tau_{w}$, Pa	Maximum Shear Stress Magnitude τ, Pa	PVL Flow Rate, kg s^−1^	Volume of Region with Stress > 300 Pa, mm^3^	Residence Time in Region with Stress > 300 Pa $t_{300}$, µs
PVL I (7.9 mm^2^, 24.3 mm^3^)	0.71	0.090	1203	1048	0.0338	0.145	4.56
	0.73	0.219	1251	1088	0.0345	0.187	5.75
	0.75	0.348	1256	1093	0.0345	0.248	7.61
	0.78	0.439	1180	1031	0.0332	0.256	8.16
	0.84	0.348	1121	983	0.0321	0.170	5.61
	0.88	0.219	1081	949	0.0314	0.134	4.51
	0.92	0.090	1057	929	0.0311	0.116	3.95
PVL II (1.5 mm^2^, 4.9 mm^3^)	0.71	0.090	973	804	0.0052	0.072	14.67
	0.73	0.219	1021	839	0.0053	0.081	16.17
	0.75	0.348	1044	856	0.0054	0.087	17.29
	0.78	0.439	1013	834	0.0052	0.077	15.60
	0.84	0.348	939	780	0.0050	0.062	13.27
	0.88	0.219	879	735	0.0048	0.053	11.70
	0.92	0.090	853	715	0.0047	0.050	11.21
PVL III (18.5 mm^2^, 56.4 mm^3^)	0.71	0.090	687	628	0.0816	0.335	4.35
	0.73	0.219	1185	1043	0.0805	0.317	4.18
	0.75	0.348	1156	1018	0.0803	0.467	6.16
	0.78	0.439	1095	969	0.0776	0.463	6.33
	0.84	0.348	1042	928	0.0751	0.316	4.46
	0.88	0.219	1041	925	0.0737	0.263	3.78
	0.92	0.090	700	636	0.0734	0.253	3.66
PVL IV (5.0 mm^2^, 15.8 mm^3^))	0.71	0.090	1132	899	0.0194	0.164	8.97
	0.73	0.219	1189	944	0.0200	0.186	9.90
	0.75	0.348	1208	963	0.0201	0.198	10.43
	0.78	0.439	1169	935	0.0197	0.196	10.55
	0.84	0.348	1087	867	0.0188	0.160	9.01
	0.88	0.219	1018	814	0.0182	0.141	8.21
	0.92	0.090	989	791	0.0180	0.135	8.00
